# Evaluating Acute Bilateral Foot Drop: A Case Report

**DOI:** 10.7759/cureus.32794

**Published:** 2022-12-21

**Authors:** Arsh N Patel, Colby Kihara, Carter Gay, Katie Oakley, P.J. Reddy

**Affiliations:** 1 Department of Research, Alabama College of Osteopathic Medicine, Dothan, USA; 2 Department of Internal Medicine, Decatur Morgan Hospital, Decatur, USA

**Keywords:** drop foot, foot drop, pes cavus deformity, common peroneal neuropathy, spinal cord compression weakness, spinal cord compression, neuropathy, bilateral foot drop

## Abstract

We illustrate the case of an 84-year-old Caucasian female who presented with complaints of bilateral lower extremity weakness and ambulation difficulties complicated by a unilateral deep venous thrombosis. Physical examination on hospital admission revealed an acute onset of bilateral foot drop with pes cavus deformity. Bilateral foot drop has been associated with a more chronic presentation due to metabolic, neurologic, and musculoskeletal etiologies. Acute onset of bilateral foot drop has been poorly defined in the literature and is considered a rare pathologic phenomenon, requiring additional investigation into the underlying cause of the presentation. We hypothesize that a spinal cord compression at the T12-L1 level resulted in L5 nerve root compression, resulting in our patient’s presentation. Definitive treatment has not been established for this condition; however, studies have been completed to evaluate surgical versus conservative approaches to help restore patients' ambulatory function. Our aim is to incorporate this case report into the limited current literature on acute bilateral foot drop as well as outline possible treatment methods to restore impaired functionality.

## Introduction

To date, the occurrence of foot drop in the global population has not been well established in the literature. The worldwide incidence has not been reported, and a significant national online survey showed that just 17% of US respondents are familiar with the term, although the parameters of what was "familiar" were not well defined [1]. Foot drop is defined as an inability to dorsiflex and evert at the ankle joint due to weakness of the tibialis anterior muscle but may also include weakness of the extensor hallucis longus and extensor digitorum longus. Weakness is defined as a muscle strength test that results in anything less than 3/5 according to the Medical Research Council (MRC) Scale for Muscle Strength; for example, when the foot can no longer be actively lifted against gravity [2]. Patients suffering from foot drop will often complain of dragging their toes, difficulty walking, climbing stairs, or frequent falls. Patients may also experience associated low back pain, posterolateral thigh pain, and/or numbness in the leg or foot [3].

The most common cause of unilateral foot drop is lumbar disc herniation or foraminal stenosis at the L4-L5 level of the lumbar spine. Bilateral foot drop with acute onset can result from many etiologies, including injury to the deep fibular or sciatic nerves, a compressive neuroma, muscular dystrophy that affects both tibialis anterior muscles, central nervous system pathologies (multiple sclerosis, stroke, amyotrophic lateral sclerosis), and even rapid intentional weight loss [4]. It is also important to note that some cases of acute bilateral foot drop may have a hereditary component, such as Charcot-Marie-Tooth disease [5]. However, most conditions of foot drop are related to chronic or progressive conditions rather than an acute onset of presentation.

Here we discuss the case of an 84-year-old female who presented with complaints of bilateral lower extremity weakness that resulted in impaired ambulation. During the examination, the presence of acute bilateral foot drop and pes cavus was discovered. We hypothesize that a T12-L1 compressed spinal cord found on our patient’s MRI may have led to the bilateral compression of the L5 nerve root, causing acute bilateral foot drop. Our purpose in presenting this interesting case is to increase the limited literature on acute bilateral foot drop as well as to support the hypothesized theory behind the etiology of the presentation outlined.

## Case presentation

An 84-year-old Caucasian female with a known history of hypothyroidism, controlled with 75 micrograms (mcg) of levothyroxine; hypertension, controlled with 20 milligrams (mg) of lisinopril; polyneuropathy treated with 25 mg of amitriptyline; vitamin B12 deficiency controlled with oral supplementation of 1000 mcg; and ovarian cancer status post total hysterectomy, presented to the outpatient clinic for a follow-up encounter, complaining of difficulty with ambulation for the past two months in addition to recent weakness in both lower extremities, which rendered her wheelchair-dependent. She denied any history of tobacco or alcohol use. Incidentally, the patient also presented with asymmetric left lower extremity swelling. An outpatient venous Doppler ultrasound revealed a popliteal deep venous thrombosis (DVT), most likely attributable to her recent immobility. Due to the abrupt nature of her ambulatory troubles and the secondary complication of DVT with an elevated risk of pulmonary embolism, she was prompted for direct admission to undergo further neurologic evaluation and the initiation of anticoagulation therapy.

On hospital admission day one, the patient revealed no subjective complaints; however, physical examination showed bilateral pes cavus deformity with foot drop, 2+ patellar reflexes, 1+ Achilles reflexes, and a negative plantar response. The patient had 0/5 muscle strength in dorsiflexion and eversion and 3/5 strength in plantarflexion and inversion, according to the Medical Research Council (MRC) Scale for Muscle Strength. Her only symptom was lower extremity weakness, with no interference with sensory, bladder, or bowel function. In order to rule out metabolic causes of bilateral foot drop, laboratory studies were performed to evaluate hypothyroidism; an MRI of the brain was performed to rule out intracranial pathology; an MRI of the cervical-thoracic-lumbar spine was ordered to rule out spinal cord pathology.

**Figure 1:**
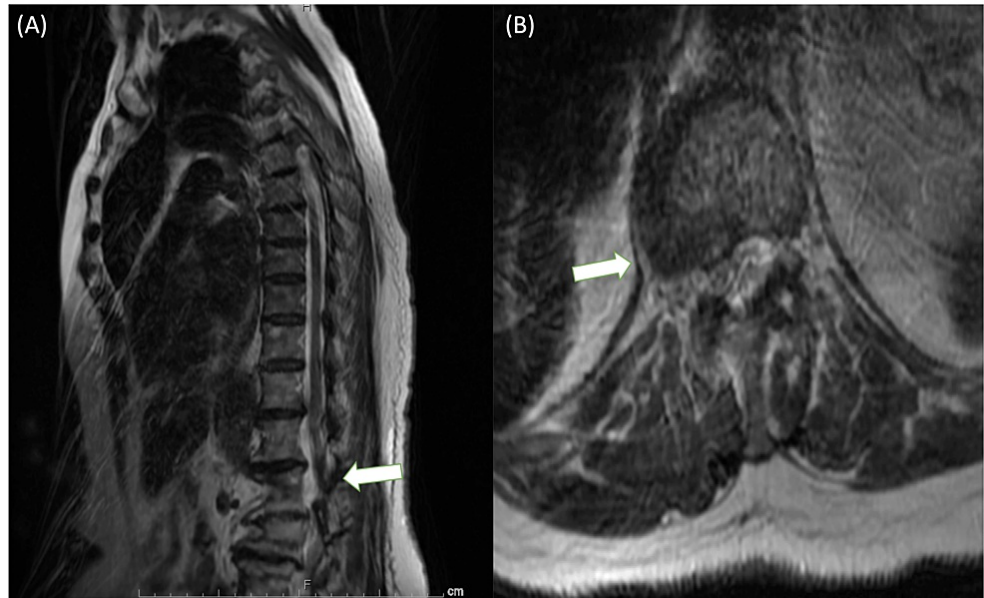
MRI of the thoracic spine (A) Sagittal T2-weighted MRI reveals cord compression at the T12-L1 level. (B) An axial T2-weighted MRI view reveals lateral disc bulging causing cord compression at the T12-L1 level.

**Figure 2:**
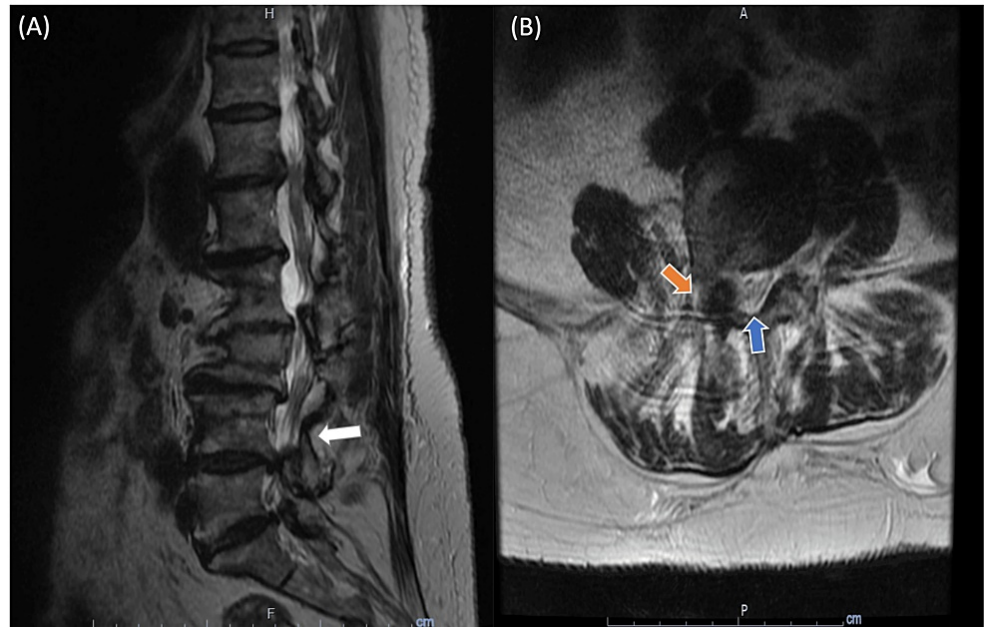
MRI of the lumbar spine (A) Sagittal T2-weighted MRI of the lumbar spine shows the L4-L5 level. (B) An axial T2-weighted MRI shows severe spinal stenosis and foraminal narrowing (orange arrow) with ligamentum flavum hypertrophy (blue arrow) at the L4-L5 level.

Coagulopathy studies were negative for factor V Leiden, antiphospholipid antibodies, and the prothrombin G202010A mutation; however, homocysteine levels were elevated at 15.7 micromoles per liter (umol/L), which prompted in-hospital five-milligram folic acid supplementation intravenously in 0.9% normal saline. Thyroid-stimulating hormone levels were elevated at 9.92, but free thyroxine (T4) levels were elevated in outpatient labs, which resulted in a dose adjustment to the present 75 mcg dosage of levothyroxine. Vitamin B12 levels were elevated at 1006 picograms per microliter (pg/mL), making subacute combined degeneration unlikely. Initial D-dimer was elevated at 2.36, and the patient was started on enoxaparin 70 mg subcutaneously every 12 hours for five days. Brain magnetic resonance imaging (MRI) revealed chronic ischemic microvascular changes with no evidence of acute intracranial pathology. Thoracic and Lumbar MRI revealed cord compression in the T12-L1 level and severe spinal stenosis with foraminal narrowing in the L4-L5 level, respectively (Figure 1 and Figure 2). 

On hospital admission day two, a neurology consultation was requested. Neurology evaluated and requested the patient be transferred for neurosurgical consultation; however, the patient declined and was started on 4 mg of intravenous dexamethasone to reduce inflammation, edema, and pain, preventing further neurologic damage. Additionally, the patient developed a Klebsiella urinary tract infection and was treated appropriately with 500 mg of levofloxacin intravenously in 100 mL of dextrose 5% in water (D5w) for three days. Hospital admission days three to five were uneventful, as the patient reported subjective improvement in symptoms but showed a continued absence of objective improvement in muscle strength, or talipes equinovarus with pes cavus. On hospital day five, the patient was discharged with a foot-ankle bracing orthosis, home health, rivaroxaban, and an outpatient neurosurgical consult for additional evaluation. The patient returned to the clinic two weeks after discharge, presenting with no significant change in reflexes but with a minimal improvement of 1/5 muscle strength in dorsiflexion and eversion. The neurosurgeon determined that she was not a surgical candidate. 

## Discussion

Foot drop is defined as the inability to dorsiflex and evert at the ankle joint due to weakness of the tibialis anterior muscle but may also include weakness of the extensor hallucis longus and extensor digitorum longus. The most common cause of foot drop is lumbar disc herniation or foraminal stenosis at the L4-L5 level of the lumbar spine, causing unilateral presentation. The case explored involves a patient who presented with two months of muscle weakness and sudden onset ambulation difficulty, which led to complications of deep vein thrombosis in the left lower leg. During the evaluation, an acute bilateral foot drop and moderate pes cavus were noted. Her acute presentation warranted a thorough workup to explore possible metabolic, neurologic, and musculoskeletal causes. Overall, there is limited data in the current literature presenting acute bilateral foot drop. Similar to a case presented by Kertmen et al., we discussed that a T12-L1 compressed spinal cord found on our patient’s MRI may have led to bilateral compression of the L5 nerve root, causing the acute bilateral foot drop in our patient [6].

**Figure 3:**
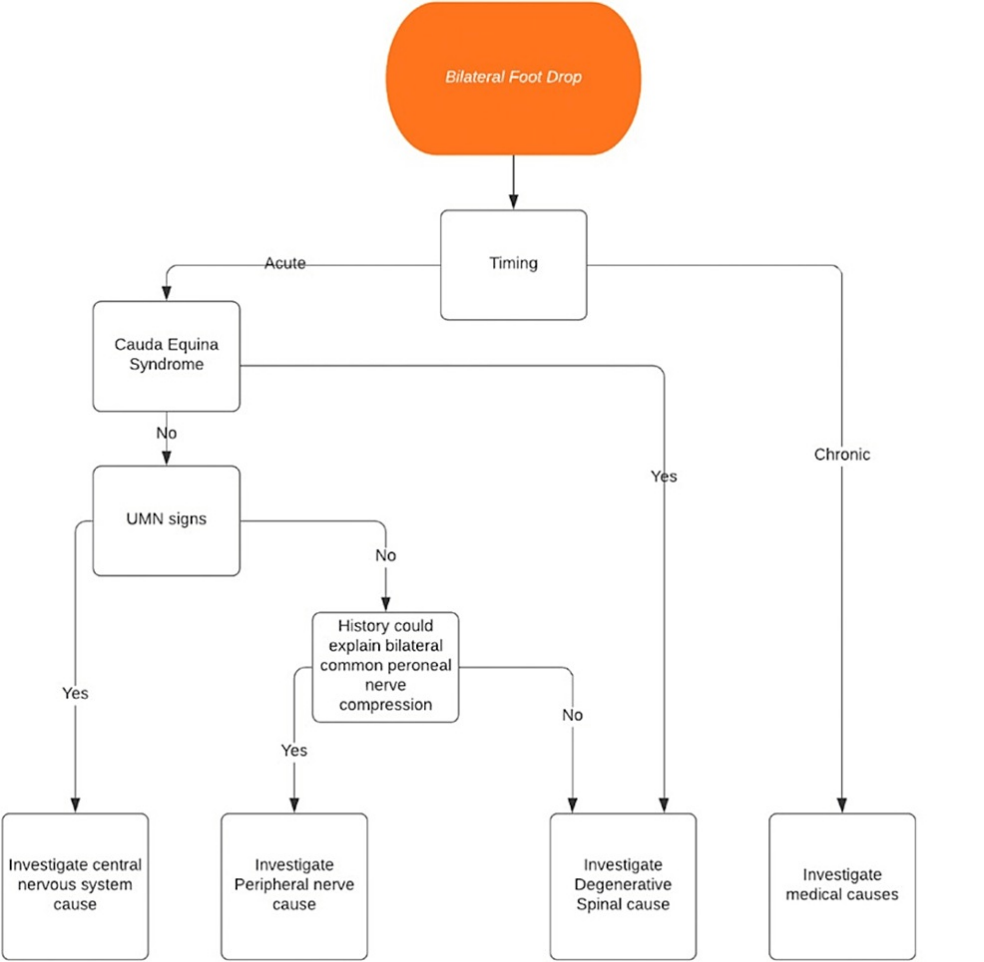
A flow chart of a proposed evaluation for investigating bilateral foot drop presentation Source: [9] License: The original article is distributed under the terms of the Creative Commons Attribution 4.0 International License (http://creativecommons.org/licenses/by/4.0/), which permits unrestricted use, distribution, and reproduction in any medium, provided you give appropriate credit to the original author(s) and the source, provide a link to the Creative Commons license, and indicate if changes were made*. * Permission was obtained and no changes were made to the original image.

The thoracolumbar junction is anatomically composed of the spinal cord, epiconus, conus medullaris, and cauda equina. The complexity of the thoracolumbar region may result in multiple non-specific neurologic symptoms, including both upper and lower motor neuron lesions. A compressive lesion at the T12-L1 level of the spine is believed to cause foot drop as a non-specific neurologic sign; however, bilateral root paralysis is a rare pathologic condition that requires further investigation to correctly manage and treat the patient [7]. Electrophysiological examination plays a key role in determining the spinal causes of foot drop from peripheral causes [8]. The suggested workup for acute bilateral foot drop has been outlined by Demetriades et al. (Figure 3) [9].

Treatment for the underlying cause of acute bilateral foot drop has been poorly defined in the literature. The decision to pursue surgical intervention is dependent on the patient’s degree of impaired dorsiflexion, surgical risk score, and willingness to undergo extensive surgery. There has been no clinical trial to date that specifically quantifies the efficacy of surgery versus conservative therapy using foot-ankle bracing orthoses for clinical improvement [8,2]. In case a patient fails conservative therapy and the risk of undergoing spinal surgery is far too great, Carolus et al. outline an alternative surgery using tendon transfer to obtain approximately 30% of strength restoration and a significantly improved quality of life [2]. Peroneus splints are the mainstay of conservative therapy, but they present with the disadvantages of abnormal gait patterns, cosmetic insecurities, and pressure points due to insufficient adaptation to individual foot shape [2]. Concurrent physical therapy may help maintain the range of motion in the ankle and knee joints, as well as preserve gastrocnemius muscle tone using gait training. Additionally, patients with advanced dorsiflexion weakness may benefit from functional electrical stimulation (FES) devices. FES devices have been suggested to improve natural movement, and a randomized controlled trial by Kottink et al. (p=0.010) showed an increase in ambulatory speed of 20% compared to controls using the six-minute walking test [10,11].

There have been multiple studies on chronic bilateral foot drop, most of which result from conditions such as, but not limited to, anorexia nervosa, Crohn’s disease, myasthenia gravis, anterior horn cell disease, Guillain-Barré syndrome, and myopathies [10]. Sudden onset of bilateral foot drop is a rare occurrence, and patients should be evaluated for cauda equina syndrome, bilateral foraminal stenoses, and an anterior communicating artery intracranial aneurysm [9].

In patients with acute bilateral foot drop, a thorough work-up is warranted to determine the underlying cause. Our hypothesis suggests that a compressed spinal cord in the T12-L1 region may cause bilateral compression of the L5 nerve roots, which led to acute-onset foot drop. There is limited literature to date regarding a tethered cord at the T12-L1 spinal level and bilateral foot drop, so this case report intends to add to the existing literature. A consideration of the limited literature on acute bilateral foot drop may be attributed to under-reporting by providers. A multidisciplinary team, including neurology, neurosurgery, internal medicine, and orthopedics, should be consulted to determine the underlying cause; the most optimal treatment method should then be identified per the patient’s wishes and stratified surgical risk.

## Conclusions

Bilateral foot drop with acute onset is a unique and complex clinical presentation that requires a thorough approach to diagnosis and treatment. Due to their emergent nature, it is important to rule out permanently disabling or deadly causes of bilateral foot drop, including cauda equina syndrome and aneurysm of the anterior cerebral artery. Other less critical causes include bilateral foraminal stenosis and disc herniation. Although rare, spinal cord compression occurring at the T12-L1 level with resultant compression of the L5 nerve root is an etiology to consider in patients with bilateral foot drop. The existing literature on this link is limited and remains unclear, but this case report may serve to strengthen that evidence. Overall, management of bilateral foot drop will depend on its determined etiology and often necessitate a multidisciplinary approach.
